# Combating mosquito-borne diseases using genetic control technologies

**DOI:** 10.1038/s41467-021-24654-z

**Published:** 2021-07-19

**Authors:** Guan-Hong Wang, Stephanie Gamez, Robyn R. Raban, John M. Marshall, Luke Alphey, Ming Li, Jason L. Rasgon, Omar S. Akbari

**Affiliations:** 1 Division of Biological Sciences, Section of Cell and Developmental Biology, University of California, San Diego, CA USA; 2 Division of Epidemiology and Biostatistics, School of Public Health, University of California, Berkeley, CA USA; 3 Innovative Genomics Institute, Berkeley, CA USA; 4 Arthropod Genetics, The Pirbright Institute, Pirbright, UK; 5 Department of Entomology, The Pennsylvania State University, University Park, PA USA; 6 The Center for Infectious Disease Dynamics, The Pennsylvania State University, University Park, PA USA; 7 The Huck Institutes of the Life Sciences, The Pennsylvania State University, University Park, PA USA; 8 Present Address: State Key Laboratory of Integrated Management of Pest Insects and Rodents, Institute of Zoology, Chinese Academy of Sciences, Beijing, China

**Keywords:** Animal breeding, CRISPR-Cas systems

## Abstract

Mosquito-borne diseases, such as dengue and malaria, pose significant global health burdens. Unfortunately, current control methods based on insecticides and environmental maintenance have fallen short of eliminating the disease burden. Scalable, deployable, genetic-based solutions are sought to reduce the transmission risk of these diseases. Pathogen-blocking *Wolbachia* bacteria, or genome engineering-based mosquito control strategies including gene drives have been developed to address these problems, both requiring the release of modified mosquitoes into the environment. Here, we review the latest developments, notable similarities, and critical distinctions between these promising technologies and discuss their future applications for mosquito-borne disease control.

## Abstract (web summary)

Mosquito-borne diseases pose significant global health burdens. In this review, the authors explore Wolbachia and genome engineering approaches to mosquito-borne disease population control.

## Introduction

Roughly half of the world’s population is at risk of mosquito-borne diseases, with the highest-burden for socioeconomically disadvantaged populations. Urbanization, globalization, climate change, and land-use shifts have each contributed to the re-emergence and expansion of mosquito-borne diseases. For example, dengue incidence has increased >30-fold in the past 50 years and outbreaks of chikungunya, yellow fever, and malaria have increased in size and frequency since 2014. The 2015–2016 Zika virus (ZIKV) epidemic in Latin America and the Caribbean also resulted in hundreds of thousands of infections, resulting in large-scale socioeconomic disruptions. Supply-chain disruptions due to the coronavirus disease 2019 pandemic are expected to increase the number of malaria-related deaths in sub-Saharan Africa in 2020–2021 as well.

There is a critical need for safe, sustainable approaches to reduce the burden of mosquito-borne pathogens. Common mosquito control strategies with chemical insecticides and environmental management are only moderately effective, in part due to resistance arising from physiological (e.g., insecticide resistance) and behavioral changes (e.g., mosquitoes change their blood-feeding times in response to bed nets). Chemical interventions also have unintended effects on non-target insects, including pollinators.

**Fig. 1:**
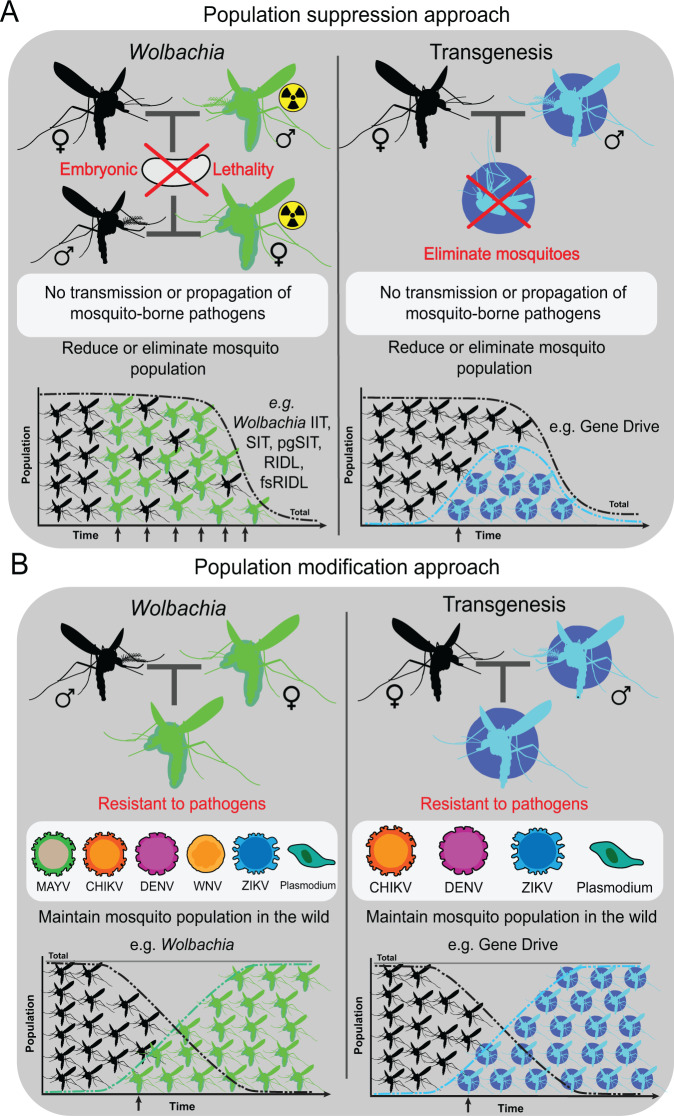
*Wolbachia* and transgene-based approaches for mosquito population suppression and population modification. **A** *Wolbachia* and transgene-based approaches for population suppression. *Wolbachia*-infected males can suppress mosquito populations through CI effects in the early embryo. To prevent fertile *Wolbachia*-infected females from escaping the sex-sorting step, an irradiation step is included to render them sterile. Using transgene-based approaches, mosquitoes can be engineered to induce lethality in the immature or adult stage of the life cycle. In suppression approaches, reducing the number of mosquitoes will result in reduced pathogen transmission. **B** *Wolbachia* and transgene-based approaches for population modification. Several studies have demonstrated the pathogen-blocking capabilities of *Wolbachia*. This feature can be used to modify mosquito populations for pathogen resistance. As *Wolbachia-*infected females also have reproductive manipulation advantages (due to CI), pathogen blocking can spread throughout wild mosquito populations. In transgene-based approaches, strategies can be designed to inhibit replication of a specific pathogen through the desired mechanism (RNAi, over-expression of innate immune pathways, etc.). When linked to a gene drive, these strategies could possibly spread throughout mosquito populations. Both *Wolbachia* and transgene-based approaches seek to maintain the mosquito population. Arrows represent mosquito releases. The multiple arrows in the *Wolbachia* IIT approach indicate that multiple releases are needed to achieve suppression. For simplicity, the SIT, pgSIT, RIDL, and fsRDIL approaches are mentioned as examples in panel A due to their requirement of multiple releases. These approaches do not utilize *Wolbachia*, despite being under this category in the figure. MAYV mayaro virus, CHIKV chikungunya virus, DENV dengue virus, WNV West Nile virus, ZIKV Zika virus.

Recently there has been an expansion in the development of genetic control technologies, involving modified mosquitoes designed with the goal to achieve either population suppression (Fig. 1A), or population modification (Fig. 1B). Suppression strategies include the sterile insect technique (SIT), incompatible insect technique (IIT), and various transgene-based technologies including gene drives. In population modification strategies, pathogen-resistant (“refractory”) mosquitoes are designed to be released into wild populations, where they can spread their heritable modifications to prevent pathogen transmission. Examples include the use of the heritable pathogen-blocking *Wolbachia* (Box 1) and various transgenic technologies. In this review, we compare and contrast mosquito control interventions aimed at either population suppression, or modification, highlighting recent developments in the use of *Wolbachia*-infected mosquitoes and transgenic strategies.

Box 1 *Wolbachia* induced cytoplasmic incompatibility (CI). *Wolbachia*-infected females transmit *Wolbachia* to their offspring via egg cytoplasm. If mated to *Wolbachia* uninfected males the incompatibility between the sperm and egg (unidirectional CI) results in non-viable offspring. In the presence of two *Wolbachia* strains, pairings between *Wolbachia*-infected females and uninfected males or males infected with a different *Wolbachia* strain (bidirectional CI) leads to the death of offspring. Viable offspring are generated from *Wolbachia*-infected females when mated with uninfected males or males infected with the same *Wolbachia* strain, but *Wolbachia*-infected males only produce viable offspring when mated with females infected with the same *Wolbachia* strain.

## Wolbachia-based approaches for mosquito control

*Wolbachia* is an intracellular reproductive parasite of arthropods and nematodes, naturally found in many insect species. Transmitted vertically from mother to offspring, *Wolbachia* maximize their transmission by manipulating host reproduction through feminization, parthenogenesis, male-killing, cytoplasmic incompatibility (CI), and by increasing host fitness with or without reproductive manipulation^1^. Through CI, *Wolbachia*-infected females produce viable *Wolbachia*-infected offspring when mated with uninfected males, or males infected with the same *Wolbachia* strain. However, *Wolbachia*-infected males only produce viable offspring when mated with females infected with the same *Wolbachia* strain (see figure in Box 1). Thus, although males are essentially dead ends for *Wolbachia* transmission, they can reduce the reproductive output of uninfected females, giving *Wolbachia*-infected females a relative reproductive advantage and enabling spread into populations. Interestingly, some important vector species, including *Aedes aegypti*, are naturally free of *Wolbachia*. Therefore, to use *Wolbachia* for control, a *Wolbachia* strain must be heritably introduced to establish a *Wolbachia*-infected colony.

## Wolbachia-based population suppression

*Wolbachia* IIT is a population suppression approach whereby male mosquitoes infected with *Wolbachia* are released into a wild population lacking that *Wolbachia* strain. Mating between *Wolbachia*-infected males and wild females results in nonviable offspring. Multiple male-only releases over time can suppress/eliminate mosquito populations and potentially interrupt disease transmission (Fig. 1A). Ideal strains for *Wolbachia* IIT should have high penetrance of sterility in matings between *Wolbachia*-infected males and wild females (ideally 100%) and should ensure similar mating competitiveness between *Wolbachia*-infected and wild males. Several *Wolbachia* strains that satisfy these conditions have been successfully transfected into *Aedes*, *Anopheles*, and *Culex* mosquitoes.

Despite advances, several drawbacks of IIT limit its long-term sustainability. IIT requires multiple, frequent releases of large numbers of perfectly sex-sorted male mosquitoes. The effort and resources required to implement IIT on a scale needed to disrupt disease transmission may not be sustainable for many areas. Accidental, unintended releases of *Wolbachia*-infected females into the population can compromise IIT as the *Wolbachia*-infected females are fertile with both infected and uninfected males (Box 1) and therefore their offspring inherit the infection. Over time as *Wolbachia* spreads, *Wolbachia* becomes more abundant, even fixed, rendering the *Wolbachia* strain obsolete for IIT due to compatibility between infected females and released males. In fact, recent *Wolbachia* IIT trials in Singapore to suppress populations of *Ae. aegypti* were compromised by the rapid establishment of the *Wolbachia* strain in the field resulting from accidental releases of even an extremely small fraction of females (estimated that only three females were released)^2^. Notably, this study employed the most advanced sex sorting technologies available today^3^, yet still fell short of the sex sorting efficiencies necessary to avoid *Wolbachia* establishment in the population^2^. These results suggest that *Wolbachia* IIT technologies alone will unlikely be fruitful for long-term, sustained population suppression, particularly in smaller populations where the unintentional release of fewer *Wolbachia* infected mosquitoes can lead to more rapid IIT establishment in the population. If IIT strains do become established in populations, however, their pathogen blocking properties may still reduce disease transmission and therefore remain beneficial for disease control programs (see “*Wolbachia*-based population modification”).

To overcome the establishment of the *Wolbachia* IIT strain, the Singapore trial also used a combined IIT and low-dose radiation sterilization approach to achieve sustained population suppression^2^. This combined IIT-SIT approach was also previously implemented in a small field trial with *Aedes albopictus* (Fig. 1A)^4^ and ensures that unintentionally released *Wolbachia*-infected females are sterile, preventing maternal transmission of *Wolbachia* to their offspring and maintaining the fidelity of the strain. While this radiation-based sterilization process can compromise fitness, it has been used for many years in SIT programs, whereby radiation is used to generate sterile offspring through the generation of chromosomal damage or lethal mutations. Notwithstanding, there remain critical questions as to whether this approach is ideal due to the impacts of radiation on fitness and possibly vector competence^5^. Post irradiation vector competence was not addressed in either study, but radiation treatment could increase the vector competence of released females by impacting the density or transmission of *Wolbachia*, or by inducing mutations in the *Wolbachia* itself, which could possibly affect its pathogen blocking capabilities or other natural immune functions in the vector^5^. When mosquito populations are already infected with another strain of *Wolbachia*, which is the case for *Ae. albopictus*, for example, it is likely unnecessary to couple IIT and SIT. This is due to bidirectional CI, whereby both directions of a cross are incompatible (Box 1), which can successfully suppress populations while retaining high male fitness and low vector competence^5^. The long-term sustainability of this combined IIT and SIT approach is further convoluted by the lack of data on post-intervention population recovery. Certainly, combining the two approaches will also increase the costs associated with implementing these technologies. In particular, the reduced fitness of the released *Wolbachia*-infected irradiated males^2^ may increase the quantity and frequency of releases needed to achieve significant population suppression. These considerations, along with more long-term and larger-scale data, are critical to evaluating the sustainability and scalability of these combined approaches for effective disease control.

## *Wolbachia*-based population modification

Population modification approaches to disease control aim to modify vector populations to harbor heritable factors that reduce or block pathogen transmission. Remarkably, *Wolbachia* has been demonstrated to naturally reduce transmission of multiple arboviruses (i.e., dengue, West Nile, chikungunya, Zika, and Mayaro) and even the malaria parasite, *Plasmodium*^6–8^. Studies have suggested that *Wolbachia* may block pathogens by competing for fatty acids, regulating host microRNAs, or upregulating innate immune response pathways^9^, or may interact directly with viral RNA to limit pathogen infection, dissemination, and transmission^10^. The precise mechanism for the anti-pathogen transmission activity of *Wolbachia* remains unclear but is hypothesized to be due to either *Wolbachia*-associated activation of host immunity or competition between the virus and *Wolbachia* for cellular resources^11^. Both of these mechanisms could interfere with virus replication, but these interactions are complicated, varying by the host, *Wolbachia* strain, and pathogen.

Field trials of *Wolbachia*-based population modification in Australia demonstrated that *Wolbachia* infection rates reached up to 90% at 11 weeks following an initial release of *Wolbachia*-infected female mosquitoes^12^ and successfully reduced the dengue transmission in Cairns and Townsville cities^13^. High *Wolbachia*-infection rates were sustained for 6 months following release in dengue virus (DENV)-endemic Yogyakarta, Indonesia, where *Wolbachia*-infected adults or eggs were released over 20 or 24 weeks, respectively^14^ and, importantly, resulted in a significant reduction in dengue incidence in the treatment area^15,16^. High *Wolbachia*-infection rates (>80%) were also sustained for more than 2 years after release in DENV-endemic Kuala Lumpur, Malaysia^17,18^ and recently in Brazil, where significant reductions in dengue, Zika, and chikungunya incidence were also observed in *Wolbachia* intervention areas^19^. In general, *Wolbachia*-based population modification strategies appear to require fewer releases than IIT strategies and allow the release of both sexes of *Wolbachia-*infected mosquitoes, enabling long-term persistence in the environment (Fig. 1B). While these efforts are exciting, how evolutionarily stable are the *Wolbachia* pathogen blocking characteristics? What happens when either the virus, mosquito, or *Wolbachia* itself evolves over time—will the pathogen blocking abilities break down—if so how will this be resolved? These are just a minority of open questions that deserve future exploration.

Box 2 Gene drives biased inheritance of genetic modifications. By normal Mendelian inheritance, a transgenic allele would be expected to be inherited at a 50% frequency. Gene drives, on the other hand, convert alleles heterozygous for the transgene to homozygous, thereby increasing the allele frequency of the transgene to >50%, and sometimes >90% in the offspring harboring the most efficient gene drive designs. Gene drives not only serve as a way to more rapidly introduce transgenes into a population, but due to the fitness costs of transgenes and positive selection for the wildtype allele, they are likely necessary to maintain most transgenes in a population as well. Without gene drives, most transgenes would simply not reach a high enough population frequency to make a significant impact on disease transmission.

### Transgenic approaches for mosquito control

#### Gene drive

The “selfish” biased transmission by *Wolbachia* has a similar outcome to that of a gene drive (GD), also “selfish”, but a genetic-based system that can spread through populations by biasing inheritance in its favor^20,21^ (see figure in Box 2). While varying dramatically in their mechanisms^20,22^, GDs can selfishly enable their spread without necessarily conferring a selective advantage to their carriers^23^. This aspect is important for the genetic control of mosquitoes, as mosquito-borne pathogens generally have a little adverse effect on infected mosquitoes. Thus, refractory genes, which impart resistance to pathogens, are unlikely to confer a significant fitness advantage to their carriers. Although inundative releases may be sufficient for some purposes^24^, refractory genes generally need to be linked to GDs for large-scale dissemination and persistence.

Scientists are developing synthetic GDs, which are often mechanistically inspired by natural GDs (e.g., *Medea*, homing endonucleases) but developed from scratch, allowing them to be better understood and tailored for specific pathogens/vectors. There are several GD types with different characteristics including homing-based gene drives (HGDs)^25–27^ and sex-linked meiotic drives^28,29^, which have been demonstrated in mosquitoes, other GD types include *Medea* and various under dominance systems^30–32^. In CRISPR HGDs^20,33^, CRISPR-associated protein 9 (Cas9) is guided by a programmable guide RNA (gRNA) to generate a double stranded break (DSB) in a precise location. This DNA break is then repaired using the cell’s homology-directed repair (HDR) machinery. When this process occurs in the germline of heterozygous individuals, wild-type alleles get converted into drive alleles, thereby enabling super-Mendelian inheritance of the drive allele.

**Fig. 2:**
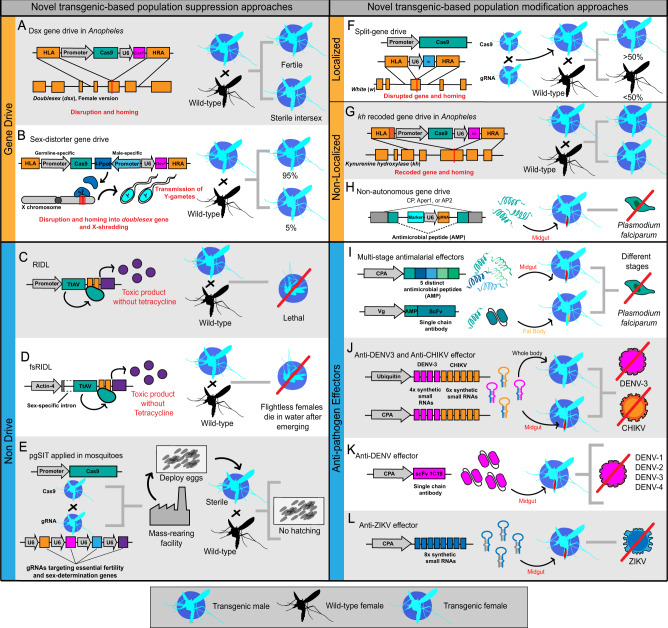
Examples of novel suppression and modification approaches in transgenic mosquitoes. Illustrations of recently developed population suppression approaches that utilize unique components to achieve mosquito suppression. **A** Gene drive (GD) suppression approach for *Anopheles* mosquitoes, which takes advantage of the sex determination pathway to produce fertile males and sterile females^42^. **B** Sex-distorter GD programmed to home into *dsx* and express an endonuclease that shreds the X-chromosome^43^. High sex-bias ratios towards males enable a population crash after sufficient generations. **C** RIDL, a self-limiting approach, consists of a dominant lethal gene that utilizes modified components of the Tet-OFF operon system^47, 48^. In the absence of tetracycline, transactivator (TtaV, green) binds to the operon sequence (orange) to induce toxic product expression in a tissue- and temporal-specific manner. High concentrations of toxic products will lead to lethality. **D** fsRIDL, a similar approach to RIDL, with added sex-specificity. A sex-specific intron ensures that TtaV protein will express only in flight muscles of females to prevent them from flying^47, 48^. **E** Potential application of pgSIT in mosquitoes. Transgenic mosquitoes carrying components encoding Cas9 and several guide RNAs (gRNAs) targeting sex-determination genes will enable the production of sterile male offspring^49^. **F** Self-limiting split drive^25^. Separating both Cas9 and gRNA/GD element components enables a safe, noninvasive, self-limiting system. **G** Recoded GD prevents fitness load associated with disrupting two copies of *kh* gene^53^. **H** Non-autonomous GD designed to have minimal components is used to produce an antimicrobial peptide in mosquito midgut to inhibit *Plasmodium* in these tissues^26^. **I** Multistage effector transgenes with the capacity to target several life stages of *Plasmodium*^61^. Transgene containing five antimicrobial peptides is expressed after a blood meal. In another configuration, a single-chain antibody linked to an antimicrobial peptide was effective. **J** Transgenes produce microRNAs to induce the RNAi pathway of mosquitoes to target and inhibit dengue virus serotype 3 (DENV-3) and chikungunya virus (CHIKV) replication and transmission^62^. **K** Anti-DENV transgene expresses an engineered single-chain antibody to confer resistance to four DENV serotypes^63^. **L** Anti-Zika virus (ZIKV) transgene uses eight synthetic small RNAs to induce the RNAi pathway against ZIKV^64^.

Despite aspirations that HGDs can solve world health issues, there are safety concerns due to the predicted ability of HGDs to persist indefinitely and invade non-target populations^34^. To address this, scientists have proposed various types of “confinable” [or “local”] gene drives whose properties allow them to be restricted to the vicinity of the release site(s), and sometimes also to limit their temporal persistence. For example, scientists have proposed the use of homing-based gene drive (split HGD) as an alternative to autonomous HGD^25,35–37^ (see Fig. 2F). In an autonomous drive, a single unit comprising Cas9 and guide RNA (gRNA) is inserted at a target location^20,33^. Split drives separate the CRISPR components across two or more genetic loci, with at least one component unable to drive. Large-scale releases can allow a split HGD to reach high frequencies and persist long-term in the target population^25^. However, limited introductions (e.g., accidental release^36^ or migrants invading non-target populations) cannot reach high frequencies, because the non-driving component is limited and declines from a low initial frequency due to negative selection. In this way, split HGDs enable spatiotemporal confinement of HGD elements^25^ and may provide an optimal platform for a safe phased release program of a gene drive. Split HGDs have reduced spread relative to equivalent autonomous drives, especially at low prevalence; however, this issue can be substantially restored by linking several elements together if desired^38^. Recent advances enabled the development of a split HGD in an *Aedes* mosquito^25,27^. Li et al. examined drive dynamics of a GD element comprising a gRNA targeting a phenotypic gene, *white*, together with an unlinked source of germline Cas9. Researchers achieved inheritance rates of up to 94%. This proof-of-principle study paves the way for further development of linked effectors for population modification strategies or transgenes useful for population suppression in *Ae. aegypti*.

#### Population suppression

Several studies have demonstrated HGD suppression systems in *Anopheles* mosquitoes^39–43^. An HGD was designed to target female fertility genes of *An. gambiae*, to decrease both female reproductive output and mosquito population size^39^. In another example, an HGD targeting *doublesex (dsx)* eliminated laboratory cage populations of *An. gambiae*^42^ (Fig. 2A). This system was designed to prevent resistant-allele accumulation at the target site, which is an issue for CRISPR-based GDs, by taking advantage of the highly conserved nature of *dsx*. Resistance alleles are generated when non-HDR DSB repair mechanisms create germline insertions or deletions (indels) at the target site^44,45^. These sites can then become resistant to drive cleavage, and as these indels accumulate over time due to increased imperfect repair events and/or positive selection of the resistance allele, they will lead to extinction of the GD from the population^46^. To attempt to further incrrease the stability of the drive, an alternative GD design was engineered by incorporating a previously characterized X-chromosome shredding nuclease, I-PpoI, into the CRISPR-based *dsx* GD system^43^ (Fig. 2B). Experiments revealed a biased sex ratio towards males and the eventual collapse of a small laboratory cage population. These innovative studies demonstrate the versatility of new technologies to overcome previous limitations. Given that these suppression drives have not been trialed in the field to date, it’s unclear whether these systems will be stable enough to face the existing natural variation and expected evolution of resistance in the environment and persist and function long enough to suppress and eliminate a target species.

Non-GD, genetic-based technologies have demonstrated population and disease suppression in smaller-scale field trials. The release of Insects carrying a Dominant Lethal gene (RIDL) has been successfully used for many years for the control of insect pests, including mosquitoes^47,48^ (Fig. 2C, D; Table 1). Despite successes with RIDL, costs associated with mass-rearing using tetracycline and fitness costs associated with the initial strains have motivated researchers to innovate other non-GD technologies and RIDL strains with lower fitness costs. An alternative, non-GD approach for mosquito control, precision-guided sterile insect technique (pgSIT), takes advantage of efficient CRISPR-mediated biallelic lethal/sterile mosaicism to produce sterile males and dead/intersex females^49^. Initially demonstrated in fruit flies, this approach circumvents the fitness costs associated with SIT (i.e., radiation-associated costs) and RIDL (i.e., tetracycline-associated costs). The pgSIT study characterized three dual-gRNA strains, all targeting the *beta-2-tubulin* gene and one of three sex determination genes: *sex lethal*, *transformer*, or the female isoform of *dsx*. When these dual gRNA strains were crossed to three Cas9 strains, male progeny were sterile, and females were either dead or converted to sterile intersex. Importantly, pgSIT males were able to compete with wild-type males for females. This approach provides an exciting opportunity for application in mosquitoes, because genes like *beta-2-tubulin*, and *dsx,* are conserved in mosquitoes (Fig. 2E). Recently, this system was adapted to *Ae. aegypti*^50^ by leveraging U6 promoters for gRNA expression^25^ targeting essential genes and available Cas9 strains^51^. This technology works during early embryogenesis, enabling the release of eggs (rather than fragile adult mosquitoes) into the field. Given that sterile pgSIT males can be generated and scaled in a factory then released, neither naturally existing polymorphisms in the gRNA target sites, nor the evolution of resistance in the wild, are expected to impact this approach, as they would for gene drives, and therefore this technology may prove to be more evolutionarily stable and effective.

**Table 1:** Comparison between *Wolbachia* and transgene-based approaches.

	Population modification			Population suppression			
	*Wolbachia*	HGD	*ClvR*	IIT (*Wolbachia*)	HGD	RIDL	pgSIT
Proof-of- principle in mosquitoes?	Yes		No	Yes			
Confinable?	Depends on fitness costs	Depends on GD type		No, releases of females can result in establishment and spread^2^	Depends on GD type	Yes	
Capacity to genetically engineer multiple strains	No	Feasible		No	Feasible		
Reversible?	No	Depends on GD type		No, releases of females can result in establishment and spread^2^	Depends on GD type	Yes	
Field releases or large cage studies	Australia^12^, Malaysia^17^, Indonesia^14, 15^, Vietnam^94^, Colombia^69^, Brazil^19^	None		China^4^, USA^3^, Singapore^2^, Thailand^7^, Burma^8^	Large cage assessments for a Sex-distorter^95^	Brazil^96^, Cayman Islands^97^, Panama^98^, USA^77^	None
Release frequency	Low	Depends on design/ fitness		Very High	Depends on design/fitness	High	
Mechanism of evolving resistance?	Temperature, host physiology, either pathogen, Wolbachia, or the mosquito can evolve resistance	Mutations in CRISPR machinery, NHEJ^a^ events, natural polymorphisms at the target site(s), effector gains nonfunctional mutations, pathogen evolves resistance		Temperature, host physiology/fixation of Wolbachia strain by accidental releases of females	Mutations in CRISPR machinery, natural polymorphisms at a target site, NHEJ events	Second-site suppressor of zygotic lethality	Mutations in CRISPR machinery in rearing facility; contamination of rearing strains

^a^NHEJ nonhomologous end-joining.

#### Population modification

Using synthetic, or naturally occurring, effector genes to reduce pathogen transmission is an alternative approach for mosquito-borne disease control. One concern with population suppression strategies is that eradicating mosquito populations may lead to continuous reinvasion from neighboring populations, or other species that occupy the same ecological niche. Population modification approaches may provide a sustainable and cost-effective means of maintaining local elimination of pathogen susceptible mosquitoes (e.g., malaria, dengue) while providing a barrier to prevent such reinvasion, but there are nuances to carefully consider for even this intervention. For example, population modification requires the spread of an evolutionary stable gene drive linked to an anti-pathogen effector with demonstrated stability and effectiveness against wild/evolving circulating pathogens. In 2015, a population modification approach was developed using a HGD linked to an antimalarial effector in *Anopheles stephensi*^52^, achieving super-Mendelian inheritance of the effector gene, however, both fitness costs and drive resistant alleles immediately appeared limiting the utility of this HGD. Recently, a recoded HGD rescue system in *Anopheles stephensi* was developed to relieve the fitness costs associated with a nonfunctional target site, and prevent the formation of resistance alleles, and this system showed promising performance in multi-generational laboratory population cages^53^ (Fig. 2G). This system could be further improved by redesigning the drive to target an essential gene and providing a recoded rescue within the drive. In fact, this architecture termed Home and Rescue (HomeR) has been recently demonstrated in flies with high transmission rates and low resistant allele generation rates, and robust performance in population cages^54,55^. Another study demonstrated an effector-linked HGD using minimal genetic modifications in malaria mosquitoes^26^ (Fig. 2H). Proof-of-principle experiments revealed that an effector construct containing homology arms to an endogenous gene and an artificial intron (gRNA and fluorescent marker) within the effector resulted in 99% of individuals inheriting the HGD. This study provides an exciting alternative approach towards designing HGDs with minimal components to decrease associated fitness costs and increase the drive efficiency of anti-pathogen effectors.

Additional nonhoming-based designs for modification include toxin-antidote CRISPR GD systems, such as “cleave and rescue” elements (*ClvR*)^56,57^ and “TARE” drives^58^, which can enhance their transmission by creating conditions in which progeny lacking these systems perish. These function using CRISPR to disrupt the function of an endogenous essential gene, and encode a linked cleavage-resistant copy of the target gene that can provide rescue to inheriting individuals. These nonhoming toxin-antidote GD designs are able to spread and overcome resistance-related challenges akin to homing-based drive architectures. Although *ClvR* has only been demonstrated as a proof-of-principle in fruit flies, this GD has the potential to become a promising strategy for future mosquito modification approaches.

To date, effectors have been engineered to overexpress endogenous transcription factors from innate immune pathways (i.e., Toll, IMD, and JAK-STAT), or to express synthetic effectors, such as single-chain antibodies, antiviral hammerhead ribozymes, and small RNAs that target mosquito-borne viruses via the host RNA interference (RNAi) pathway^59,60^. Many early iterations of these effectors, however, were limited in their ability to target multiple pathogens, or parts of the pathogen life cycle. Recently engineered strategies to address these critical issues have included multistage effector transgenes against different life stages of *Plasmodium falciparum* in *A. stephensi*^61^ (Fig. 2I), a dual-antiviral effector targeting two distinct viral families^62^ (Fig. 2J), and an anti-DENV effector against four genetically distinct DENV serotypes^63^ (Fig. 2K). Strategies against arboviruses such as ZIKV^64^ provide insights into how modification approaches could be adapted to re-emerging mosquito-borne pathogens (Fig. 2L). Notwitstanding these developments, while each of these effectors has been developed and rigorously tested in the laboratory, it remains to be determined how these will perform in complex ecosystems with variable environmental conditions when exposed to greater genetic diversity and diverse evolving microbial communities—will they be effective and for how long? Moreover, is it possible that these effectors could generate a bottleneck and thereby force the evolution of more virulent/pathogenic strains^60^? Given these concerns, significant future work needs to be undertaken to test their efficacy and stability when challenged with pathogens that are presently circulating and evolving in the environment^60^, prior to any release of such effectors linked to a GD into the environment.

### Comparing *Wolbachia* and transgenic approaches

While at the forefront of innovation for mosquito-borne disease control, *Wolbachia* and transgenic approaches have distinct similarities and differences (Table 1). Particularly due to the extensive genetic toolbox currently available, transgenic-based approaches are flexible, optimizable, and permit designs of creative control approaches as compared to *Wolbachia-*based approaches which cannot be engineered. Transgenic approaches have been successfully applied to many mosquito species, whereas the success of *Wolbachia* transinfection and the stable transmission seems to be somewhat species-dependent (e.g., successful in *Ae. aegypti* and *A. stephensi* but difficult in *An. gambiae*). Gene editing tools also enable precise genetic changes that permit creative control approaches^51^. These tools can facilitate the development of GDs with varying degrees of spatiotemporal spread, persistence, and novel traits, although they are yet to be rigorously tested outside the lab^20,65^. In contrast, *Wolbachia* engineering has not been successful, thus *Wolbachia*-based methods depend on the inherent properties of the *Wolbachia* strains that are found in nature.

Optimization capabilities of pathogen resistance also vary by approach. *Wolbachia* strains are capable of blocking many arboviruses^6,8,66^, but they can do so only through mechanisms naturally dictated by the bacterium, which are not well understood, nor manipulatable. Transgenic approaches can be tailored to specific pathogens through various mechanisms (RNAi, immune pathways, etc.). This mechanistic aspect is an important design consideration, as it influences the ability to target pathogens at different developmental stages or multiple sites, which can be crucial for preventing or mitigating the emergence of pathogen resistance^60,61^. To date, there is no evidence of pathogen escape, or evasion, in *Wolbachia-*infected wild populations, but there have been numerous examples in laboratory studies^67^ with different pathogens and different arthropod hosts underscoring the importance of continued monitoring for such an escape. For example, an elegant artificial selection and gene association study on *Wolbachia-*infected mosquitoes collected from the WMP program release site in Cairns, Australia, was able to quickly select pathogen blocking phenotypes and identified 61 genes associated with modulation of pathogen blocking, suggesting that breakdown of pathogen blocking could occur easily under some circumstances^68^. If pathogen evasion did arise in wild populations, it may be difficult to modify this technology to address it or to remove the system from the environment.

Both approaches are likely to become established at a high frequency in relatively short time scales. Field releases of *Wolbachia*-infected mosquitoes have repeatedly demonstrated successful *Wolbachia* spread and effective pathogen blocking into populations on a scale ranging from 6 months to >2 years^15,16,18,19,69^. High temperatures may reduce CI and transmissibility of some *Wolbachia* strains though, which may impact their establishment and persistence in some field locations^70^. Theoretical modeling suggests that some HGD systems can be established in a population within a year of the initial release^25,71^. There is no current field data on HGDs, however, to validate these models.

The production and scaling cost considerations for these technologies may also differ. In general, the scaling costs are going to be lower for technologies that require lower release frequencies (Table 1). For these approaches, costs are generally going to be inversely related to persistence and confinability—the longer the system will persist and the farther it can spread—will generally result in lower production and scaling costs. Therefore, using *Wolbachia* for population modification will require less production and scaling costs than for *Wolbachia* IIT. For gene drive, non-confinable HGDs for either population modification, or suppression, would have less production and scaling costs than confineable split-HGDs which would require repeated releases to control a local population.

Some *Wolbachia* strains can block pathogen transmission, enabling release without sex-sorting, with the intent of *Wolbachia*-based population modification (i.e., using the selfish inheritance properties of *Wolbachia*). Notwithstanding, under certain laboratory conditions, *Wolbachia* strains have also been observed to enhance infections^72–74^, but it is not yet clear whether this phenomenon is applicable for stable *Wolbachia* infections released into the wild. In field demonstrations, however, *Wolbachia* was able to quickly invade wild populations and reduce pathogen transmission^12,16,19^. Given that evolution of either the virus, mosquito, or *Wolbachia* itself may result in alterations to the *Wolbachia* transmission blocking abilities—future efforts to characterize these long-term effects in wild populations where *Wolbachia* has been deployed should be of very high priority. Genetic control systems are intended to be transmitted only from parents to offspring (“vertical transmission”). The possibility of horizontal transmission to non-target species, followed by spread within that species, has been widely discussed but seems highly implausible.

Regulatory hurdles and public perception differ substantially between the two methods. Although mosquitoes transfected with *Wolbachia* are clearly modern biotechnological products, they have not encountered the same regulatory hurdles as transgenic approaches and go through different regulatory pathways^75^ (e.g., *Wolbachia*-based approaches are considered “veterinary chemical products” in Australia). This lack of regulatory clarity is an issue for genetically and non-genetically modified methods in many jurisdictions. Early engagement with communities, stakeholders, and the public has led to fewer public-relation barriers for *Wolbachia*-based approaches^76^. Both *Wolbachia* population suppression (IIT) and modification approaches have been successfully trialed in several countries. The same is true for transgenic suppression approaches; the RIDL approach successfully obtained regulatory and community approval in several countries, despite the more complex environment for genetically engineered organisms. Multiple trials in Brazil, Panama, and the Cayman Islands showed strong suppression of target *Aedes* mosquito populations. Some RIDL trials, however, have received strong public opposition, notably the trials in Key Haven, FL, which delayed trials in this area until recently^77^ while *Wolbachia*-based approaches have received essentially no opposition^78^. GD technologies have received significant opposition, but the lessons learned from GE have served GD programs, such as Target Malaria, and they have developed a meticulous, cautious step-wise phased approach towards a potential approval for transgenic GD releases. As transgene-based GD field releases have yet to become a reality, however, time will tell whether this seemingly more judicious approach to field trial approval meets expectations.

Finally, a remediation plan to recall these technologies is also essential. Both approaches have the possibility of losing function, for example, loss of transgene expression or pathogen evolving resistance to the linked effector^60^; or for *Wolbachia* loss of CI^79^, or reduction/loss of pathogen refractoriness which is vulnerable to environmental factors, temperature, and host diet^80^. Moreover, remediation may be necessary if these approaches are affected by an unintended consequence, shift in public opinion, or end of a trial period. Resistance alleles can limit transgene spread for GDs. However, innovative GD designs, such as reversal GDs that recall a problematic GD from the population^20,81,82^, have been proposed to address this problem^46,83,84^. Anti-CRISPR proteins can theoretically be applied as “natural brakes” to CRISPR-based HGDs^85^. Ongoing field studies indicate that *Wolbachia* can remain at high infection frequency with strong pathogen blocking and CI abilities for up to 8 years from initial invasion^12^. However, there is no simple way to remove *Wolbachia*-infected mosquitoes after release and spread. One could perhaps release mosquitoes infected with a different *Wolbachia* strain and exploit bidirectional incompatibilities between the two strains, replacing the old strain with the new one^86^. Alternatively, one could super-infect the old mosquito strain with an additional *Wolbachia* strain to generate a new strain that can spread into the already-invaded population. However, this scenario can lead to superinfection, where mosquitoes with multiple *Wolbachia* strains can have an incomplete maternal transmission or incompatible CI^87^. The release of wild-type mosquitoes to dilute the *Wolbachia* strain to sub-threshold levels is another potential remediation strategy; however, such wild-type releases would include large numbers of wild-type female mosquitoes capable of disease transmission and are therefore not ideal. Finally, IIT-SIT could be used to reduce the population below the threshold needed for persistence. If the populations are susceptible to insecticides, insecticide-based tools could also be used for remediation for some GD and *Wolbachia* technologies.

### Concluding remarks and future perspectives

*Wolbachia* and transgene-based tools are both innovative approaches that may revolutionize mosquito*-*borne disease control. Immense progress has been made in genetically modified and *Wolbachia*-infected mosquitoes, leading to field trials around the world. Despite appreciable progress, knowledge gaps remain regarding *Wolbachia*-mosquito and *Wolbachia-*pathogen interactions. For example, not much is known about environment-host interactions, or how the host microbiome affects *Wolbachia* efficiency in mosquitoes^74,88^. Additional work is also needed to optimize CI and pathogen-blocking capabilities. Screening for temperature-insensitive *Wolbachia* strains is also crucial to avoid CI loss^79^. Identification and characterization of CI-inducing genes can pave the way for alternative control strategies. Likewise, additional work on transgene-based strategies is required, including reducing the cost of transgene fitness, finding ideal target sites for GD insertion, and eliminating resistance-allele formation, determining the effectiveness of anti-pathogen effectors using pathogens present in the field. For sterile male-based suppression approaches, male mosquitoes must be released multiple times. Improvements in mass mosquito production, precise sex separation, and release technologies are crucial to make these approaches more sustainable and cost-efficient^3^.

Ethical and regulatory issues over GD use, including the role of public participation in GD development^89^, informed consent, regulation^90^, possilbe use of global registries, and associated risks, should be carefully considered before any implementation^91–93^. Discussions regarding who should regulate and assess the risks of GD technology are in process and may take years to reach a consensus. The concept of a phased GD release approach is likely the best option to enable safe testing of the various components beginning with confinable approaches first. For example, to safely test GD components (e.g., CRISPR/Cas9) one could begin with a self-limiting type approach (e.g., pgSIT) to suppress the local population (Phase 1). This could be followed by a confinable split-GD approach which would enable the safe testing of linked effectors (Phase 2). Then if even necessary, this could be followed by a non-confinable GD approach—which would use the same components (e.g., gRNAs, Cas9, target genes, linked effectors, etc.) that were tested in the previous phases (Phase 3).

As *Wolbachia*-based population control technologies do not result in the genome modification of the target species, they do not have the notable stigma associated with genetic-based technologies. To date, *Wolbachia*-based control efforts have therefore been an easier technology for the public to accept. Several countries, including Australia, Malaysia, Indonesia, Vietnam, Colombia, and Brazil have already released *Wolbachia*-infected mosquitoes, with some observing reductions in local mosquito-borne disease transmission (Table 1). Regardless of the approaches used (whether transgenic or *Wolbachia*), scientists should scrutinize all proposed technologies to fully understand their advantages and disadvantages. There is no single silver bullet for mosquito disease control, and different communities may prefer different approaches that suit their local needs. Therefore, the development of multiple approaches is crucial.

Going forward, the prospect of controlling mosquito-borne diseases using innovative technologies is promising and we are in the golden age of the development of population control technologies. With increasing public confidence, time, and progress, we will continue to see these technologies developed and safely used to tackle global health issues and safe human lives.
